# Genomic and Epidemiological Evidence of a Dominant Panton-Valentine Leucocidin-Positive Methicillin Resistant *Staphylococcus aureus* Lineage in Sri Lanka and Presence Among Isolates From the United Kingdom and Australia

**DOI:** 10.3389/fcimb.2019.00123

**Published:** 2019-04-26

**Authors:** Sharla M. McTavish, Sarah J. Snow, Ellie C. Cook, Bruno Pichon, Sarah Coleman, Geoffrey W. Coombs, Stanley Pang, Cesar A. Arias, Lorena Díaz, Emma Boldock, Steve Davies, Mangala Udukala, Angela Marie Kearns, Sisira Siribaddana, Thushan I. de Silva

**Affiliations:** ^1^Healthcare Associated Infections and Antimicrobial Resistance AMR Division, National Infection Service, Public Health England, London, United Kingdom; ^2^The Florey Institute for Host-Pathogen Interactions and Department of Infection, Immunity and Cardiovascular Disease, University of Sheffield, Sheffield, United Kingdom; ^3^Department of Microbiology, Sheffield Teaching Hospitals NHS Foundation Trust, Sheffield, United Kingdom; ^4^Antimicrobial Resistance and Infectious Diseases Research Laboratory, School of Veterinary Life Sciences, Murdoch University, Murdoch, WA, Australia; ^5^Center for Antimicrobial Resistance and Microbial Genomics and Division of Infectious Diseases, UTHealth, McGovern Medical School, Houston, TX, United States; ^6^Center for Infectious Diseases, UTHealth School of Public Health, Houston, TX, United States; ^7^Molecular Genetics and Antimicrobial Resistance Unit, International Center for Microbial Genomics, Universidad El Bosque, Bogota, Colombia; ^8^Anuradhapura Teaching Hospital, Anuradhapura, Sri Lanka; ^9^Faculty of Medicine and Allied Sciences, Rajarata University of Sri Lanka, Mihintale, Sri Lanka; ^10^Department of Medicine, Wright Fleming Institute, Imperial College London, London, United Kingdom

**Keywords:** methicillin resistant *Staphylococcus aureus*, Panton-Valentine leucocidin, Sri Lanka, CC5, whole genome sequencing

## Abstract

**Objective:** To undertake the first detailed genomic analysis of methicillin-resistant *Staphylococcus aureus* (MRSA) isolated in Sri Lanka.

**Methods:** A prospective observational study was performed on 94 MRSA isolates collected over a 4 months period from the Anuradhapura Teaching Hospital, Sri Lanka. Screening for *mec*A, *mec*C, and the Panton-Valentine leucocidin (PVL)-associated *lukS-PV/lukF-PV* genes and molecular characterization by *spa* typing was undertaken. Whole genome sequencing (WGS) and phylogenetic analysis was performed on selected multilocus sequence type (MLST) clonal complex 5 (CC5) isolates from Sri Lanka, England, Australia, and Argentina.

**Results:** All 94 MRSA harbored the *mecA* gene. Nineteen *spa* types belonging to nine MLST clonal complexes were identified. Where origin of the sample was recorded, most isolates were from skin and soft tissue infections (70/91; 76.9%), with fewer causing bacteremia (16/91; 17.6%), empyema (3/91; 3.3%) and osteomyelitis (2/91; 2.2%). Sixty two (65.9%) isolates were PVL positive with the majority (56 isolates; 90.3%) belonging to a dominant CC5 lineage. This lineage, PVL-positive ST5-MRSA-IVc, was associated with both community and hospital-onset infections. Based on WGS, representative PVL-positive ST5-MRSA-IVc isolates from Sri Lanka, England and Australia formed a single phylogenetic clade, suggesting wide geographical circulation.

**Conclusions:** We present the most detailed genomic analysis of MRSA isolated in Sri Lanka to date. The analysis identified a PVL-positive ST5-MRSA-IVc that is prevalent among MRSA causing clinical infections in Sri Lanka. Furthermore, this clone was also found among isolates from the United Kingdom and Australia.

## Introduction

Worldwide, *Staphylococcus aureus* is the primary causative agent of community-acquired skin and soft tissue infections (SSTI) and is an important cause of hospital-associated invasive infections including bacteremia, pneumonia and endocarditis (Bell et al., 2002; David and Daum, 2010). Panton-Valentine leucocidin (PVL)-positive Methicillin Resistant *S. aureus* (MRSA) is a well-documented cause of community-associated SSTI and less commonly, life-threatening infections in immunocompetent populations. Its prevalence is thought to be increasing worldwide and multi drug resistant PVL-MRSA is emerging as a threat, particularly in the Indian subcontinent (Song et al., 2011; Shallcross et al., 2013). In many developed countries, surveillance of MRSA invasive disease, characterization of high risk MRSA clones and the investigation of suspected MRSA outbreaks are achieved through public health tracking and molecular analysis. By comparison, limited data exist on MRSA infections in low and middle-income countries. A recent report has suggested Sri Lankan hospitals have the highest prevalence of MRSA for all Asian hospitals that were included in the study (Song et al., 2011). However, information on the molecular epidemiology and spectrum of clinical disease is lacking (Corea et al., 2003; Mahalingam et al., 2014; Jayaweera and Kumbukgolla, 2017; Jayaweera et al., 2017). Consequently in our study, we report on the genomic analysis of MRSA isolated from patients admitted to a major teaching hospital in Sri Lanka.

## Methods

A prospective, observational study of sequential MRSA infections in hospitalized patients was conducted at the Anuradhapura Teaching Hospital from 30th June to 31st October 2014. This hospital serves ~1.6 million people living in the rural North Central province of Sri Lanka. All MRSA isolated from any site with a clinical infection during the 4-months period were included in the study. *S. aureus* species identification from clinical samples was confirmed by colony morphology, catalase and tube coagulase tests. Screening for methicillin resistance was undertaken via disc diffusion testing using oxacillin, incubation on Mueller-Hinton agar at 33–35°C for 24 h, and interpreted according to CLSI 2005 guidelines. Ethical approval was obtained from the Ethics Review Committee, Rajarata University of Sri Lanka. Infections were defined as community-acquired (CA) if the sample was collected <48 h from admission and hospital-acquired (HA) if collected later, based on previous studies distinguishing whether MRSA infection were likely to be acquired in community or hospital settings (Cardoso et al., 2014).

Isolates were referred to the Staphylococcal Reference Service, National Infection Service, Public Health England (PHE), Colindale, London for further analysis. Initial identification was performed using the MALDI-TOF (MALDI Biotyper®, Bruker Daltonik GmbH, Germany), followed by real-time polymerase chain reaction (PCR) for *mecA* and *lukS*-PV/*lukF*-PV genes, to determine the isolate's methicillin resistance, Panton-Valentine leucocidin (PVL) status, and *spa* typing (Frénay et al., 1996; Pichon et al., 2012).

Whole genome sequencing (WGS) on selected isolates was undertaken as previously described (Garvey et al., 2016; Lahuerta-Marin et al., 2016). Genomic DNA was extracted using the Qiagen DNA mini kit (Qiagen) and QIAsymphony instrument (Qiagen). DNA libraries were prepared with the Nextera XT kit (Illumina, Cambridge, UK) and sequenced on the Illumina HiSeq 2500 instrument (Illumina), generating 100 base paired end fragments. Sequence types (ST) were derived from WGS sequences using MOST (Tewolde et al., 2016). By mapping WGS reads against reference sequences using bowtie (Langmead et al., 2009), a broad range of loci were screened for virulence factors and the immune evasion cluster (IEC), in addition to genes and chromosomal mutations associated with antimicrobial resistance (Lahuerta-Marin et al., 2016; Sharma et al., 2016). Staphylococcal Cassette Chromosome *mec* (SCC*mec*) types were deduced based on the detection of the *mec* complex and *ccr* genes by BLAST on assembled genomes generated using SPAdes (Bankevich et al., 2012). The phylogenetic relationship between isolates was determined at the core genome level by Single Nucleotide Polymorphism (SNP) analysis using an in house pipeline (phenix; https://github.com/phe-bioinformatics/PHEnix). Sequence reads were mapped to an ST8-MRSA reference sequence (NC_007795) and SNPs were called and filtered using the Genome Analysis Toolkit v2.0 (Van Der Auwera et al., 2013). The resulting alignment was used for maximum likelihood analyses using RAxML v.0 8.2.8 (Stamatakis, 2014) under the GTRCAT model with 100 bootstraps. The best tree was drawn using the ITOL application (Letunic and Bork, 2016). The sequence data supporting the results of this article are available in the European Nucleotide Archive, under project accession number PRJEB27049.

## Results

The 94 isolates submitted for further testing were confirmed as *S. aureus* by MALDI-TOF and were *mec*A positive (Table 1). Where clinical data were available (*n* = 91), the majority of MRSA isolates (*n* = 70, 76.9%) were from skin and soft tissue infections (SSTIs), with the remainder from invasive infections, including 16 (17.6%) MRSA bacteremias (Table 1). Five deaths occurred, all in patients with bacteremia. Based on the 19 *spa* types identified, the isolates could be grouped into nine MLST clonal complexes (CC) including: CC5 (*n* = 59 isolates), CC30 (*n* = 18), CC1 (*n* = 8), CC59 (*n* = 4), and single isolates belonging to CC6, CC8, CC45, CC97, and CC101. The dominant CC5 MRSA lineage (62.7% of isolates) was comprised of six related *spa* types: t002 (*n* = 51), t010 (*n* = 4), and single isolates of t045, t1062, t5490, and t7342. Sixty two (65.9%) isolates were PVL positive, the majority (56 isolates; 90.3%) belonging to the CC5 lineage. Where clinical details were available, the CC5 PVL-positive lineage was associated mainly with HA- and CA-SSTIs (42/50; 84.0%), but was also responsible for more invasive infections (Table 1), such as bacteremia (5/50; 10.0%), empyema (2/50; 4.0%) and osteomyelitis (1/50; 2.0%). Two deaths occurred, both in patients with bacteremia. All HA-SSTIs were surgical wound infections.

**Table 1:** Genotypic, demographic and clinical characteristics of MRSA isolates from Sri Lanka.

	***spa* types (No.)**	**PVL**	**No. subjected to WGS**	**Age distribution (Years)^a^**					Clinical presentations^*^					**Hospital or community acquired (No.)**
				**<1**	**1–15**	**16–60**	**>60**	**NK**	**SSTI**	**BAC**	**EMP**	**OM**	**NK**	
CC1	t657 (*n* = 3)	+	ND	1	2				2	1				CA (2), NK (1)
	t127 (*n* = 5)	+ (*n* = 1)	ND		2	2	1		5					CA (2), HA (3)
CC5	t002 (*n* = 51)	+ (*n* = 50)	28	8	15	22	5	1	42	5	2	1	1	CA (25), HA (9), NK (17)
	t010 (*n* = 4)	+ (*n* = 3)	3			3	1		3			1		CA (4)
	t045 (*n* = 1)	+	1			1			1					CA(1)
	t1062 (*n* = 1)	+	1					1	1					CA (1)
	t5490 (*n* = 1)	+	1			1			1					HA (1)
	t7342 (*n* = 1)	–	ND			1					1			CA (1)
CC6	t304 (*n* = 1)	–	ND				1			1				CA (1)
CC8	t008 (*n* = 1)	–	ND			1			1					CA (1)
CC30	t021 (*n* = 2)	+	ND			1	1		1	1				NK (1), CA (1)
	t425 (*n* = 13)	–	ND		2	10	1		6	6			1	CA (5), HA (8)
	t15007 (*n* = 1)	–	ND			1			1					HA (1)
	t4410 (*n* = 2)	–	ND		1	1			2					HA (1), CA (1)
CC45	t465 (*n* = 1)	–	ND		1				1					HA (1)
CC59	t437 (*n* = 1)	–	ND			1			1					HA (1)
	t7028 (*n* = 3)	–	ND			2		1	1	1			1	HA (2), NK (1)
CC97	t15010 (*n* = 1)	–	ND			1				1				HA (1)
CC101	t1212 (*n* = 1)	–	ND			1			1					NK (1)

All isolates were mecA positive by PCR. SSTI, skin and soft tissue infection;

* *Hospital-acquired cases of SSTI were surgical wound infections; BAC, bacteremia; EMP, empyema; OM, osteomyelitis; CA, community-acquired; HA, hospital-acquired; ND, not done; NK, not known*.

a *Gender distribution of cases: 48 male, 40 female, 6 no data available*.

To investigate whether the CC5 PVL-positive lineage was genotypically diverse, or a single circulating clone, WGS was performed on 34 isolates selected to include both invasive (four bacteremia and one osteomyelitis) and SSTI (*n* = 29) infections, samples from both children and adults, and a diverse range of *spa* types (28 t002 and six samples from other *spa* types). As CC5 PVL-positive MRSA have been identified sporadically in England, we sought to assess the relatedness of the Sri Lankan isolates to lineage-matched isolates held in the PHE archives, which represent samples submitted to a national reference laboratory. These included isolates from patients with known travel links to Sri Lanka (10 CC5 PVL-positive MRSA isolates collected between 2005 and 2014 but not known to be linked to each other in time or place) and patients with no known travel links to Sri Lanka (79 isolates: 12 CC5 PVL-positive MRSA isolates collected between 2005 and 2015, 33 CC5 PVL-negative MRSA isolates collected between 2009 and 2016, 4 CC5 PVL-positive methicillin-sensitive *S. aureus* (MSSA) isolates collected between 2011 and 2016, and 30 CC5 PVL-negative MSSA isolates collected between 2011 and 2016). Previously sequenced CC5 PVL-positive MRSA from collaborators in Australia (*n* = 14, collected in 2015) and Argentina (*n* = 3; collected in 2003) were also included as comparators.

**Figure 1:**
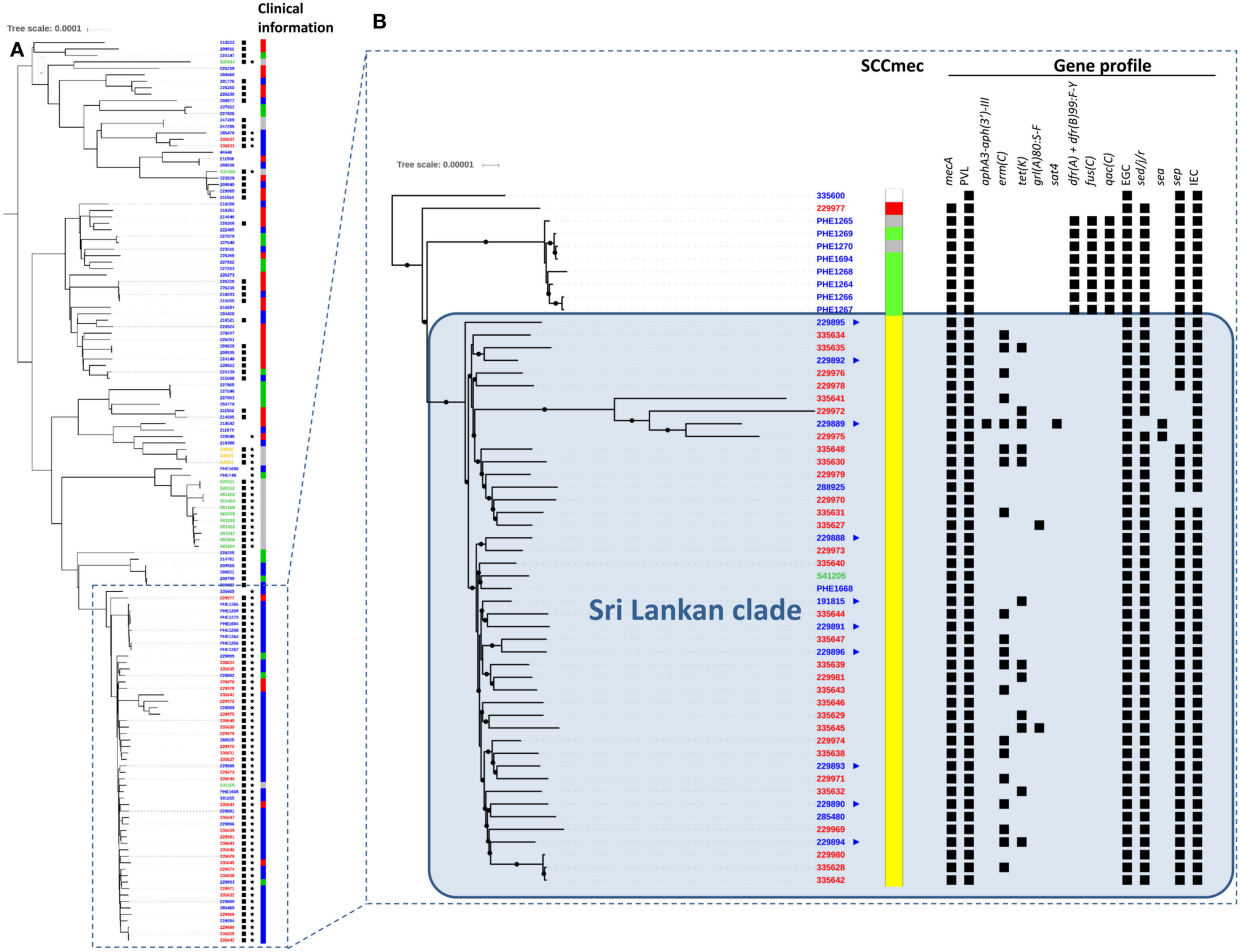
Alignment of international MLST CC5 *Staphylococcus aureus* genomes. **(A)** Unrooted phylogenetic tree indicating relationships between international CC5 *S. aureus* including their PVL (⋆) and *mecA* (■) status based on SNP analysis of whole genome sequences. Clinical information relating to each isolate is also shown (red: invasive, blue: skin and soft tissue infection, green: screening/carriage sample, gray: unknown). Phylogeny was inferred by maximum likelihood analysis using RAxML GTRCAT model with 100 bootstraps from aligned polymorphic sites allowing 20% of Ns and gaps. Polymorphic sites were called using GATK2 and filtered (AD ratio = 0.9; min depth = 10; MQ score >30; QUAL score >40) using genome NC_002745 as mapping reference. The tree was drawn using the ITOL application. Country of origin denoted as follows in sample identifiers at tips: blue: England; red: Sri Lanka; green: Australia; yellow: Argentina. Scale is in substitutions per site and indicates ≈ 130 SNPs. **(B)** 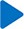 Indicates UK patients with known links to Sri Lanka. SCC*mec* types: 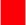 IV-a; 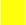 IV-c; 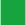 VI; 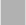 NT. In gene profile section, ■ indicates presence of gene. All sequenced *mec*A-positive isolates also contained the ß-lactamase encoding gene *bla*Z. Scale is in substitutions per site and represents ≈ 13 SNPs.

Phylogenetic analysis of CC5 strain WGS showed great variability (Figure 1A), with isolates from various countries dispersed throughout the tree. A strong geographic signal however was apparent amongst the isolates from Sri Lanka, with all but three CC5-PVL-positive MRSA isolates clustering into a single clade, herein dubbed the “Sri Lankan clade” (Figure 1B). Isolates from the United Kingdom (13) and Australia (1) were also found in the Sri Lankan clade, including three from patients with no known links to Sri Lanka. Within the clade, the isolates were identified as multilocus sequence type (ST) 5 and the MRSA isolates harbored the SCC*mec* IVc staphylococcal cassette chromosome *mec* subtype. All but one isolate encoded enterotoxin genes (*sed, sej*, and *ser*) usually found on plasmids (Fisher et al., 2018). Greater variability was apparent for other traits including the *sep* enterotoxin gene. Genes encoding resistance to erythromycin [*erm*(C)] or tetracycline [*tet*(K)] were variably detected highlighting the dynamic loss/acquisition of mobile genetic elements within the clone. Similarly, a chromosomal mutation associated with quinolone resistance (*grlA* 80:S-F) was noted sporadically. A single isolate from a UK patient with links to Sri Lanka was identified as being genotypically multi-drug resistant, encoding *blaZ, mecA, erm*(C), *tet*(K), *aphA3*, and *sat4* genes. Bayesian phylogenetic reconstruction using BEAST (data not shown) failed to provide significant temporal signal for predicting evolutionary rate and time to common ancestor.

## Discussion

Particular lineages of MRSA are frequently associated with various geographical origins e.g., ST8/USA300 (North America); ST93 (Australia); ST80 (North Africa); ST30 (South-West Pacific) (David and Daum, 2010; Chua et al., 2011). Prior to the current study, CC5 PVL-positive MRSA was reported in many countries world-wide (Monecke et al., 2011); however, their origin(s) are unclear. A recent phylogenomic study of CC5-MRSA isolates from the Western Hemisphere showed high diversity, even among strains that shared the same SCC*mec* type circulating in the same country (Challagundla et al., 2018). A study of *S. aureus* bacteremia isolates from nine Latin American countries reported that the majority of MRSA belonged to CC5 (Arias et al., 2017). Very few whole genome sequences from CC5-MRSA isolates from Asian countries are currently available.

Herein, in most instances, phylogenetic analysis of CC5 PVL-positive MRSA from four continents showed clustering according to their geographic location, suggesting they have arisen independently in different parts of world following the acquisition of PVL phage and/or different SCC*mec* elements. Our data provide evidence of a successful ST5-PVL-positive MRSA-IVc clone in Sri Lanka which also appears to be present in distant geographical regions. Thirteen isolates from England were interspersed within the Sri Lankan clade; ten with known links to Sri Lanka. One isolate from Australia also clustered within the Sri Lankan clade, however a link to Sri Lanka could not be determined.

Whilst in our study all Sri Lankan isolates were collected systematically without undue bias, it is important to acknowledge that the number of isolates is small and that they were collected from a single center over a relatively short timeframe. However, the UK CC5 PVL-positive MRSA isolates in the Sri Lankan clade, including those with known links to Sri Lanka, were collected over the course of a decade prior to our study. This suggests that wider circulation of this PVL-positive ST5-MRSA-IVc clone is likely in Sri Lanka and that our newly collected samples do not simply represent a clonal outbreak in Anuradhapura Teaching Hospital. With our current dataset, it is not possible to definitively conclude whether this clone originated from Sri Lanka and spread to the UK and Australia, or whether the origin is from another country. A larger study of isolates from other parts of Sri Lanka and globally would be required to help elucidate the origins and dissemination of PVL-positive MRSA belonging to the CC5 lineage. Travel- and migration-related acquisition and importation of MRSA strains to Europe is well-described, including a high proportion of PVL-positive isolates causing SSTI (Nurjadi et al., 2015, 2018). The ST5-PVL-positive MRSA-IVc clone identified was also responsible for both CA- and HA-infections, emphasizing the increasingly blurred lines between community and hospital-associated infections reported (Skov and Jensen, 2009). We also had limited CC5 PVL-negative MRSA isolates from Sri Lanka and did not perform WGS on these. Since PVL are carried on bacteriophages (Boakes et al., 2011), it is possible that we have missed isolates within the ST5-MRSA-IVc clade that have lost PVL. Again, a larger study is required to explore this possibility. Furthermore, such a study would allow comprehensive characterization of genetic factors important in the uptake of PVL in various CC5 clades.

In conclusion, we have presented the most detailed genomic analysis of MRSA isolated in Sri Lanka to date and have demonstrated, at least in the hospital and catchment area studied, that clinical MRSA infections in Sri Lanka are dominated by a PVL-positive ST5-MRSA-IVc clone. We have also shown the clone can be found in English patients with a history of travel to Sri Lanka. Further work is required to determine the prevalence of carriage and infection associated with PVL-positive ST5-MRSA-IVc in Sri Lanka, and the dynamics of transmission in and out of hospital, and whether these findings are replicated on a national scale.

## Ethics Statement

Ethical approval was obtained from the Ethics Review Committee, Rajarata University of Sri Lanka.

## Author Contributions

TdS, AK, and BP designed and supervised the study. SJS, EC, MU, and SS carried out the field work and initial microbiological characterization of the isolates. SC, SD, and EB carried out data analysis and interpretation of the primary dataset. SM carried out genetic characterization of isolates from LK and UK. BP performed phylogenetic analyses. SM, TdS, AK, and BP performed analysis of study data. AK, BP, TdS, and SM wrote the paper. GC, SP, CA, and LD provided data. All authors approved the final manuscript.

### Conflict of Interest Statement

The authors declare that the research was conducted in the absence of any commercial or financial relationships that could be construed as a potential conflict of interest.
